# Factors Associated With Cocaine Use Disorder: Results From the French Nationwide Repeated Cross‐Sectional OPPIDUM Study (2019–2023)

**DOI:** 10.1111/fcp.70081

**Published:** 2026-03-26

**Authors:** Zenab Alhamidi, Clémence Lacroix, Elisabeth Jouve, Thomas Soeiro, Céline Eiden, Joëlle Micallef

**Affiliations:** ^1^ Centre d'évaluation et d'information sur la pharmacodépendance – Addictovigilance Aix Marseille Univ, APHM, INSERM, Inst Neurosci Syst, UMR 1106 University Hospital, Service de Pharmacologie Clinique et Pharmacosurveillance Marseille France; ^2^ Addictovigilance Centre, Site Unique de Biologie CHU Montpellier Montpellier France

**Keywords:** abuse, addictovigilance, cocaine, dependence

## Abstract

Cocaine use has increased both globally and nationally. This trend is accompanied by a rise in clinical complications. The objective of our study was to identify factors observed to be associated with cocaine use disorder, using data from the OPPIDUM program, collecting information directly from patients with substance use disorders recruiting in care or harm reduction facilities. Cocaine users were divided into two groups: cocaine use disorder and simple use. A univariate analysis was performed to compare the groups, followed by a multivariate analysis to identify factors associated with cocaine use disorder. Between 2019 and 2023, 6863 cocaine users (28% of all OPPIDUM participants) from 116 addiction treatment centers were included. Several factors were found to be significantly associated with cocaine use disorder: extreme precariousness (OR = 1.34, 95% CI [1.12–1.61], *p* = 0.002), cocaine‐only use (OR = 1.90, 95% CI [1.45–2.50], *p* < 0.0001), alcohol dependence (OR = 1.48, 95% CI [1.30–1.68], p < 0.0001), and use of antidepressants and antipsychotics (OR = 2.08, 95% CI [1.37–3.16], *p* = 0.001; OR = 1.94, 95% CI [1.40–2.69], *p* < 0.001). These findings highlight several key factors associated with cocaine use disorder and their clinical implications. The study has limitations: potential selection bias, repeated inclusion of the same users across years, multiple modes of cocaine use, and the exploratory nature of the analyses due to uncontrolled alpha risk. Nevertheless, these insights can help healthcare professionals better understand patient profiles and provide more tailored care and prevention strategies.

**Abbreviations**
DRAMES: Décès en Relation avec l'Abus de Médicaments Et de Substances
DSM IV‐TR: Diagnostic and Statistical Manual of Mental Disorders, 4th Edition, Text Revision
FAN: French Addictovigilance Network
NPS: new psychoactive substances
OAT: opioid agonist treatment
OPPIDUM: Observation des Produits Psychotropes Illicites ou Détournés de leur Utilisation Médicamenteuse
SD: standard deviation

## 1 Introduction

Cocaine is the second most commonly used illicit drug in Europe after cannabis. In the European Union, surveys indicate that almost 2.5 million of 15‐ to 34‐year‐olds (2.5% of this age group) used cocaine in 2023 [1]. One of the greatest risks of cocaine use is the development of dependence. Five percent of cocaine users develop cocaine use disorder during the first year of use, and 20% of them will develop a long‐term cocaine use disorder [2]. The cumulative probability of transitioning to dependence is estimated at 67.5% for nicotine users, 22.7% for alcohol users, 20.9% for cocaine users, and 8.9% for cannabis users [3]. However, to date, no pharmacological treatment has been approved in the United States or Europe for cocaine use disorder. Available psychosocial approaches such as cognitive behavioral therapy and contingency management have shown limited effectiveness, although the latter appears to be the most promising [4]. In this context, recent data indicate a marked increase in hospital admissions for cocaine‐related disorders and cocaine detoxification, reflecting a growing burden on hospital‐based addiction care services [5, 6].

In France, the French Addictovigilance Network (FAN) has reported an increase in serious complications related to the use of all forms of cocaine since 2010, in parallel with an alarming increase in cocaine consumption between 2010 and 2016. This update of the national addictovigilance survey on cocaine indicates that 23% of spontaneous reports concerned cocaine. Analysis of these spontaneous reports reveals psychiatric and somatic complications (neurologic, respiratory, and cardiac) [7]. This upward trend in cocaine use also affects vulnerable populations. There was a significant increase in cocaine use among pregnant women attending addiction care centers in France, from 4.7% in 2005 to 14.3% in 2018, according to the OPPIDUM program [8]. Moreover, cocaine‐related deaths, collected via the DRAMES program, were at their highest level in 2021 and 2022 in France. The incidence of cocaine‐related deaths has risen from 4.4% in 2011 to 19.5% in 2021, and associations involving cocaine have risen from one third of reported deaths in 2011 to over half in 2021 (58%) [9].

To our knowledge, no study has comprehensively examined the factors associated with cocaine use disorder in people from substance abuse treatment facilities. Those that have been conducted were often limited to young populations or focused on co‐occurring substance use disorders [10, 11, 12]. In this context, and in response to the global and national rise in cocaine use and the severity of its consequences, this study aims to identify the factors associated with cocaine use disorder in a specific population of people with substance use disorder.

## 2 Methods

### 2.1 Data Source

The data source was the OPPIDUM program. This program is managed by the FAN. It relies on the territorial recruitment of substance abuse treatment facilities by the FAN since 1995 [13, 14]. The OPPIDUM program has consistently provided valuable insights into drug abuse monitoring [15, 16, 17, 18, 19, 20]. Each year, the data collected are entered into a centralized database after a quality control process, allowing access to a consistent and reliable dataset from the program's inception.

OPPIDUM is an annual, cross‐sectional, national, multicenter survey that collects data on substance use from users with substance use disorders. Users are interviewed in various substance abuse treatment facilities, including addiction departments (hospitalization, consultation, liaison teams), addiction treatment centers, harm reduction centers, and specialized addiction units in prisons [21, 22]. Data collection is based on anonymous questionnaires covering the following aspects: (i) sociodemographic characteristics, including age, sex, education level, and socioeconomic status (categorized as “severe precarity” for social breakdown, “precarity” for social assistance, or “regular income”); (ii) alcohol dependence; (iii) first psychoactive substance used and first substance leading to dependence; (iv) involvement in opioid agonist treatment (OAT); and (v) use of psychoactive substances in the week prior to the interview or before incarceration for prisoners. This last section includes for each drug used details on frequency, dosage, route of administration, onset of use, means of acquisition, dose escalation over the past 6 months, intended effects, concomitant alcohol use, withdrawal symptoms (both psychological and physical), and the modality of use (i.e., simple use, abuse, or dependence). Regarding the modality of use, the pattern of use was defined during the interview. This assessment is consistent with the definitions of the DSM‐IV‐TR. Indeed, users who reported use with abuse or dependence showed a significant increase in doses and reported withdrawal symptoms, which is in line with the characteristics of this category of use as defined in the Diagnostic and Statistical Manual of Mental Disorders, 4th Edition, Text Revision (DSM IV‐TR) [23] and the French Public Health Code, Article R5132‐97 [24]. More information about the OPPIDUM program has previously been published [14, 25, 26].

### 2.2 Selection of Users

Cocaine users were selected from the OPPIDUM database obtained in 2019, 2020, 2021, 2022, and 2023. We excluded users with missing data on the modality of use (*n* = 253) and those who reported both simple use and a daily frequency (*n* = 110) (i.e., probable coding error). The selected sample had similar sociodemographic and clinical characteristics to the initial. No more specific sampling strategy was managed.

We focused on data obtained in 2019, 2020, 2021, 2022, and 2023 to ensure continuity with the ongoing work of the FAN [6, 7, 27]. This timeframe was also chosen to provide recent data on cocaine users.

### 2.3 Statistical Analyses

The study size was not previously calculated. Excepted exclusions listed above, the analysis included all cocaine users who participated in the OPPIDUM survey. No statistical imputation was performed on missing values.

The characteristics of users and psychoactive substances were presented according to the cocaine consumption mode group (cocaine use disorders vs. cocaine simple use). Continuous variables were summarized by mean and standard deviation and compared using Student's *t* test. Categorical variables were summarized by number and percentage of modalities and compared using chi‐square test. Unadjusted odds ratio (OR) with 95% confidence interval were also provided. Two‐sided *p* value < 0.05 defined statistical significance. A logistic regression model was used to estimate adjusted OR for cocaine use disorders. The initial model included all the significant variables from the bivariate analysis, except for those with collinearity, of which only the most informative were retained: Alcohol dependence/concomitant alcohol use, nasal cocaine use/nasal drug use, intravenous cocaine use/intravenous drug use, new psychoactive substances use, and classic synthetic drugs use has been grouped. Age was categorized as either 40 years or ≥ 40 years. Coefficients were estimated using maximum likelihood. The same final model was achieved using both backward and forward selection, as well as stepwise selection, based on theoretical considerations. Analyses were performed using SAS Software (V9.4; SAS Institute Inc., Cary, NC, USA).

## 3 Results

**FIGURE 1:**
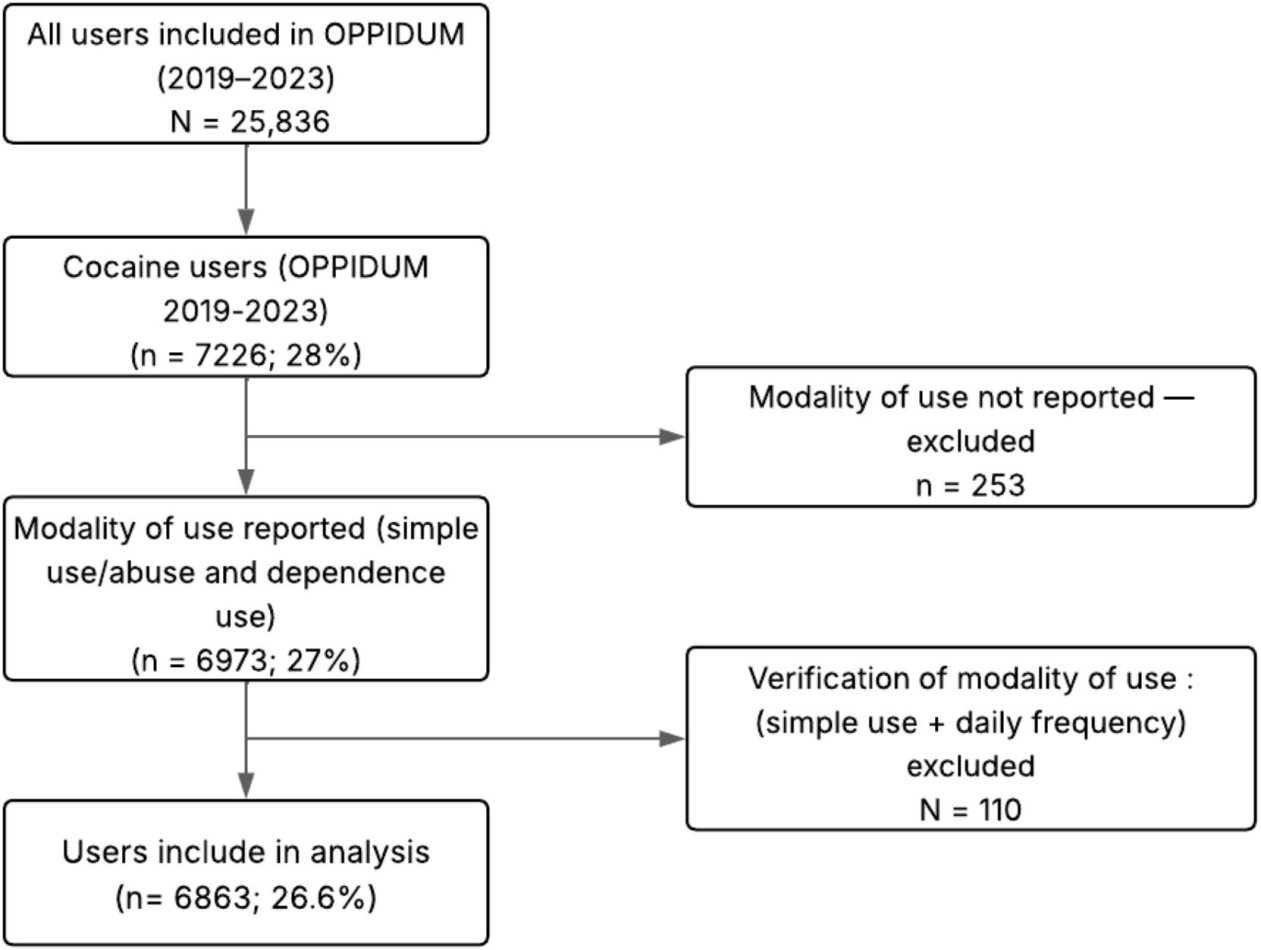
Flow diagram of inclusion and exclusion criteria applied to cocaine users from the OPPIDUM database (percentages calculated relative to the initial OPPIDUM sample [*n* = 25 836]).

**FIGURE 2:**
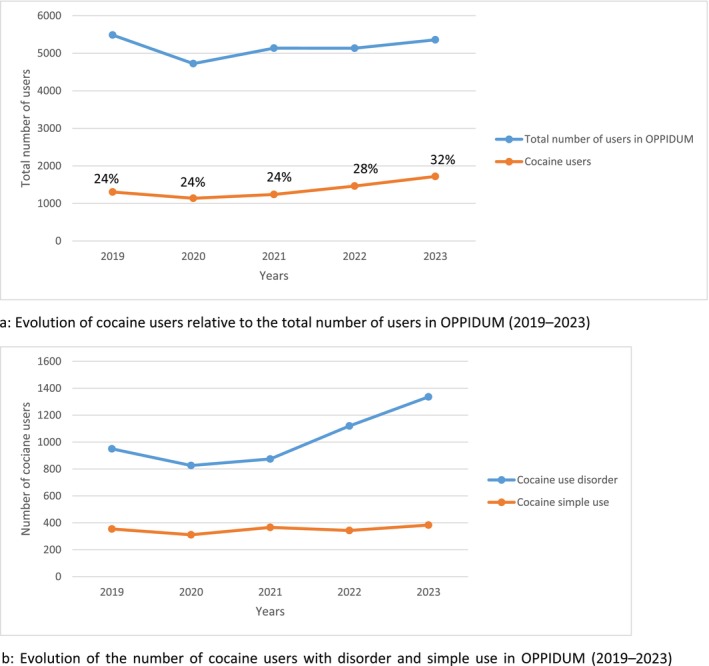
(a) Evolution of cocaine users relative to the total number of users in OPPIDUM (2019–2023). (b) Evolution of the number of cocaine users with disorder and simple use in OPPIDUM (2019–2023).

Between 2019 and 2023, 7226 cocaine users were included in the OPPIDUM program, representing 28% of the total 25 836 users. Among them, 6863 users (26.6%) were included in this analysis (Figure 1). Of these, 5106 users (74%) met the criteria for cocaine use disorder, while 1757 users (26%) were identified as simple cocaine users. The number of cocaine users varied between 23% and 32% per year during the study period (Figure 2).

### 3.1 Univariate Analysis

We compared users with cocaine use disorder (*n* = 5106) to those with cocaine simple use (*n* = 1757) in a univariate analysis (Table 1). The mean age was similar between the two groups (39.3 ± 9.5 years in users with cocaine use disorder and 38.7 ± 9.5 years in those with cocaine simple use). The majority of participants were male in both groups, accounting for 79.8% in the cocaine use disorder group and 77.5% among those with cocaine simple use.

**TABLE 1:** Univariate analysis: Comparison of users with cocaine use disorders and users with simple use of cocaine in the OPPIDUM program, using data obtained in 2019, 2020, 2021, 2022, and 2023.

	Cocaine simple use *N* = 1757	Cocaine use disorder *N* = 5106	Unadjusted odds ratio [95% confidence interval]	*p*
Age in years, mean (±SD)	39.3 (±9.5)	38.7 (±9.5)		
**Sex, *n* (%)**				
Male	1396 (79.8)	3934 (77.5)	0.87 [0.76; 0.99]	0.055
Female	353 (20.2)	1140 (22.5)		
**Socioeconomic status, *n* (%)**				
Economic resources				
Severe precarity	194 (11.4)	730 (15.0)	1.37 [1.16; 1.63]	< 0.0001
Social compensation or income	1512 (84.6)	4144 (85.0)		
**Education level, *n* (%)**				
Technical education	1039 (61.3)	2705 (55.6)	0.845 [0.76; 0.95]	< 0.0001
High school	208 (12.3)	783 (16.1)		
**Type of structure, *n* (%)**				
Harm reduction center	672 (38.2)	1037 (20.3)	0.41 [0.37; 0.46]	< 0.0001
Addiction treatment center	846 (48.1)	2906 (56.9)		
Others^a^	239 (13.6)	1163 (22.7)		
**Substances used during first experimentation, *n* (%)**				
Cannabis at first use	1260 (74.0)	3488 (71.3)	0.87 [0.77; 0.99]	< 0.032
Cocaine at first use	91 (5.3)	510 (10.4)	2.06 [1.64; 2.60]	< 0.0001
**Substance category leading to dependence, *n* (%)**				
Heroin	870 (53.5)	1832 (38.4)	0.54 [0.48; 0.61]	< 0.0001
Cannabis	420 (25.8)	1429 (30.0)	1.23 [1.08; 1.40]	< 0.002
Cocaine	157 (9.7)	1155 (24.2)	2.99 [2.99; 3.57]	< 0.0001
**Associated behaviors, *n* (%)**				
Alcohol dependence	547 (31.4)	1985 (39.5)	1.43 [1.27; 1.60]	< 0.0001
Intravenous drug use	345 (19.6)	1106 (21.7)	1.13 [0.99; 1.30]	0.073
Nasal drug use	1100 (62.6)	2826 (55.3)	0.74 [0.66; 0.82]	< 0.0001
Involved in OAT	1146 (65.3)	2740 (53.7)	0.62 [0.55; 0.69]	< 0.0001
Involved in methadone treatment	838 (47.7)	2023 (39.6)	0.72 [0.65; 0.80]	< 0.0001
Current polydrug use, *n* (%)				
Current polydrug use	1644 (93.6)	4332 (84.8)	0.38 [0.31; 0.47]	< 0.0001
Cocaine‐only users	113 (6.43)	774 (15.16)	2.60 [2.12; 3.19]	< 0.0001
Current cannabis use	774 (44.1)	1922 (37.6)	0.77 [0.69; 0.86]	< 0.0001
Current heroin use	520 (29.6)	1168 (22.9)	0.71 [0.62; 0.80]	< 0.0001
Current NPS use	198 (11.3)	403 (7.9)	0.67 [0.56; 0.81]	< 0.0001
Current Classic Synthetic Drugs use	175 (10.0)	351 (6.9)	0.67 [0.55; 0.81]	< 0.0001
Current Antidepressant use	33 (1.9)	190 (3.7)	2.02 [1.39; 2.93]	< 0.0001
Current Antipsychotic use	52 (3.0)	294 (5.8)	2.00 [1.48; 2.04]	< 0.0001
Cocaine use patterns, *n* (%)				
Concomitant alcohol use, *n* (%)	787 (45.2)	2484 (49.6)	1.20 [1.07; 1.33]	< 0.001
Route of administration of cocaine, *n* (%)				
Nasal cocaine use	923 (52.8)	2397 (47.1)	0.80 [0.71; 0.89]	< 0.0001
Intravenous cocaine use	267 (15.3)	949 (18.7)	1.27 [1.10; 1.48]	< 0.014
Way of acquisition, *n* (%)				
Donation	216 (12.5)	219 (4.3)	0.68 [0.60; 0.77]	< 0.0001
Deal	1507 (87.0)	4784 (94.8)	2.83 [2.32; 3.45]	0.014

Abbreviations: NPS, new psychoactive substances; OAT, opioid agonist treatment; SD, standard deviation.

^a^ Others: Liaison team, addictology hospitalization units, addictology consultation units, prison medical units.

### 3.2 Multivariate Analysis

In the multivariate analysis (Table 2), cocaine use disorder was associated with severe precarity (OR = 1.34, 95% CI [1.12, 1.61], *p* = 0.002), cannabis as the first substance leading to dependence (OR = 1.68, 95% CI [1.46, 1.94]; *p* < 0.0001), and cocaine as the first substance leading to dependence (OR = 3.16, 95% CI [2.59, 3.87]; *p* < 0.0001). Similarly, cocaine‐only users (OR = 1.90, 95% CI [1.45, 2.50]; *p* < 0.0001), alcohol dependence (OR = 1.48, 95% CI [1.30, 1.68]; *p* = 0.0001), antidepressant use (OR = 2.08, 95% CI [1.37, 3.16], *p* = 0.001), antipsychotic use (OR = 1.94, 95% CI [1.40, 2.69]; *p* = 0.0001), and obtaining cocaine through dealing (OR = 2.68, 95% CI [2.14, 3.36]; *p* < 0.0001) were also associated with cocaine use disorder.

**TABLE 2:** Multivariate analysis: Factors associated with cocaine use disorder in the French OPPIDUM program, using data obtained in 2019, 2020, 2021, 2022, and 2023.

	Odds ratio [95% confidence interval]	*p*
Severe social precarity vs. social assistance or income	1.34 [1.12, 1.61]	0.002
Alcohol dependence	1.48 [1.30, 1.68]	< 0.0001
Cocaine‐only users vs. poly drug use	1.90 [1.45, 2.50]	< 0.0001
Nasal use of cocaine	0.75 [0.67, 0.85]	< 0.0001
Mode of cocaine acquisition: dealing	2.68 [2.14, 3.36]	< 0.0001
Cannabis as first drug leading to dependence	1.68 [1.46, 1.94]	< 0.0001
Cocaine as first drug leading to dependence	3.16 [2.59, 3.87]	< 0.0001
Current antidepressant user	2.08 [1.37, 3.16]	0.001
Current antipsychotic user	1.94 [1.40, 2.69]	< 0.0001
Current cannabis user	0.80 [0.70, 0.91]	0.001
Current synthetic products user	0.79 [0.64, 0.97]	0.021

## 4 Discussion

The aim of our study was to identify factors observed to be associated with cocaine use disorder among users included in the OPPIDUM program between 2019 and 2023. We used data directly reported by cocaine users recruited from substance use treatment facilities across France. Several factors were associated with cocaine use disorder. These included severe precarity, alcohol dependence, and the use of antidepressants or antipsychotics. These findings reflect significant psychological and social vulnerability. The association with cocaine‐only use (OR = 1.90, 95% CI [1.45, 2.50]; *p* < 0.0001) suggests a particularly strong relationship with this substance, possibly associated with its specific effects or its central role in the individual's consumption pattern. Our findings align with the study by Liu et al. (2021), which found that polysubstance users were less likely to use cocaine heavily compared with cocaine‐only users, and were therefore at lower risk of developing cocaine use disorder [12].

Regarding the social aspect, our findings revealed that users with severe precarity were significantly more likely to present cocaine use disorder (OR = 1.34, 95% CI [1.12, 1.61], *p* = 0.002). These results are in line with national epidemiological data from the United States (2011–2015), which showed a significant increase in cocaine use among users with the lowest household incomes (< $20000), with a relative increase of over 36% and the highest prevalence in 2015 (3.12%) compared with other income groups [28]. However, other studies have reported the opposite trend, suggesting a higher risk of cocaine use disorder among users with higher incomes [3, 29]. This discrepancy may reflect changes in the cocaine market, where in France the price of a gram fell from €130 in 2012 to €90 in 2023—a 17% drop in real terms. The growing practice of selling smaller, more affordable units (€30–40 for half‐grams, or even €10–20 fractions) has also increased accessibility, particularly for low‐income users [30]. Severe precarity could also be a consequence of the addiction process itself, which increasingly centers the user's life around acquiring the substance. A broader public awareness effort is therefore essential, not only to prevent wealthier individuals from entering into dependence, but also to support more socially vulnerable users in improving their living conditions.

Then, we observed that users with alcohol dependence were significantly more numerous among those with cocaine use disorders (OR = 1.48, 95% CI [1.30, 1.68]; *p* < 0.0001). This finding is consistent with previous meta‐analyses showing that alcohol use is highly prevalent among cocaine users, with an estimated 74% reporting simultaneous use and 77% reporting concurrent use [31]. These high rates of co‐use suggest a strong behavioral and possibly pharmacological interaction between the two substances, which may contribute to the development or persistence of cocaine use disorders. This association may be partly explained by the pharmacokinetic and neurobiological consequences of concurrent alcohol and cocaine use. When consumed together, alcohol alters the metabolism of cocaine by promoting the formation of cocaethylene, a psychoactive metabolite. Cocaethylene produces similar euphoric and stimulant effects to those of cocaine and has been shown to induce conditioned place preference in animals, in a manner comparable to cocaine [31, 32, 33, 34]. The danger of this combination lies in the fact that cocaethylene has a longer elimination half‐life than cocaine—approximately 60 min—thereby prolonging stimulant effects and increasing toxicological risk. Cocaethylene is also considered more toxic to the heart and liver than cocaine itself, further compounding the health risks associated with this pattern of co‐use [35]. In addition, cocaethylene has been associated with seizures, liver damage, and impaired immune system function, and it carries an 18‐ to 25‐fold higher risk of immediate death compared with cocaine alone [36, 37]. Moreover, neuroimaging studies have further shown that users with both alcohol and cocaine dependence exhibit more pronounced structural abnormalities in white and grey matter—particularly in the frontal and temporal lobes—compared with those with alcohol use disorder alone [38, 39, 40]. These alterations include reduced white matter integrity and decreased volumes in the anterior cingulate and orbitofrontal cortices, regions critical for impulse control and decision‐making. Collectively, these findings support the hypothesis that alcohol use not only pharmacologically enhances cocaine's effects, but also exacerbates the neurobiological vulnerability to cocaine use disorder [31]. Therefore, clinicians should be particularly vigilant in informing patients about the heightened factors associated with the co‐use of alcohol and cocaine and systematically address this combination in prevention and treatment strategies.

Finally, we observed that the use of antidepressants and antipsychotics was significantly more frequent among individuals with cocaine use disorder (OR = 2.08, 95% CI [1.37, 3.16], *p* = 0.001; OR = 1.94, 95% CI [1.40, 2.69], *p* < 0.001). In light of these results, two hypotheses can be proposed. The first is the presence of psychiatric comorbidities among users with cocaine use disorder, which could justify the use of antidepressants and antipsychotics [41, 42]. The second hypothesis is the use of antidepressants and antipsychotics as part of the therapeutic management of cocaine dependence. However, the literature remains conflicting. Some studies report a potential benefit of antidepressants in the management of patients with cocaine addiction, particularly in promoting abstinence and addressing related complications such as seizures, depression, criminality, and co‐occurring psychiatric morbidity [43, 44, 45, 46, 47, 48]. However, this use is not without risks, and dose management is a critical factor to consider in order to avoid toxicity [46]. Other studies find these treatments to be ineffective and suggest that the use of antidepressants and antipsychotics is not sufficiently justified [49, 50, 51]. Some may even exacerbate certain symptoms [50, 52, 53]. Therefore, healthcare professionals should exercise caution when prescribing these medications to cocaine users. Further studies are needed to better define the usefulness, potential benefits, and limitations of such prescriptions in this specific context.

### 4.1 Limitations

The main limitation of this study is that we cannot rule out a potential selection bias (i.e., the study population may not be representative of all cocaine users seen in substance abuse treatment facilities), as the data are based on the voluntary participation. Additionally, some users may have been included more than once across different years or facilities, as the data are collected anonymously. In addition, the fact that users can report multiple modes of cocaine use (snorting, injection, inhalation, etc.) prevents us from identifying specific differences associated with a single mode of administration. Nonetheless, as the risk of alpha error inflation was not controlled, the results of this study should be considered exploratory and hypothesis‐generating, and no causal relationship can be assumed. However, to our knowledge, the OPPIDUM program has no equivalent for describing substance use in this vulnerable population [16, 17, 18], and its findings are consistent with data from other sources [1, 28, 31, 54, 55]. Another strength of this study is that, although the OPPIDUM program was not originally designed for this specific research objective, it meets the essential criteria of the study design required to answer to our research question.

## 5 Conclusion

Our study identified key factors associated with cocaine use disorder, including severe precarity, alcohol dependence, withdrawal symptoms, dose escalation, cocaine‐only use, and psychotropic medication use. These results highlight the strong psychological and social vulnerability of affected individuals and underline the need for targeted clinical attention. By clarifying the profiles most associated with cocaine use disorder, our findings may guide clinicians and policymakers toward more effective screening, prevention strategies, and ultimately the development of a dedicated clinical assessment tool.

## Author Contributions

**Zenab Alhamidi:** conceptualization; visualization; writing – original draft. **Clémence Lacroix:** conceptualization; visualization; data curation; writing – review and editing. **Elisabeth Jouve:** formal analysis; methodology; data curation; writing – review and editing. **Thomas Soeiro:** methodology; data curation; writing – review and editing. **Céline Eiden:** conceptualization; visualization; writing – review and editing. **Joëlle Micallef:** conceptualization; supervision; writing – review and editing.

## Conflicts of Interest

The authors declare no conflicts of interest.

## Data Availability

The data that support the findings of the study are available from the corresponding author upon reasonable request.
